# The Psychology of Monomaniacal Societies and Literature

**Published:** 1854-07-01

**Authors:** 


### THE JOURNAL

OF

### PSYCHOLOGICAL MEDICINE

AND

### MENTAL PATHOLOGY.

JULY 1, 1854.

### Art. I.—THE PSYCHOLOGY OF MONOMANIACAL SOCIETIES AND LITERATURE.

In a previous essay we gave a cursory consideration to a wide-spread popular delusion, which had taken the form of demonology and divination, or "the black art." We then thought it probable that, as "one fire puts out another burning," and *similia similibus curantur*, this more recent absurdity would extinguish, or at least counteract, the follies of mesmerism. In this idea we were mistaken; for, just as a monomaniac converts all current ideas into food for the delusive ideas which actuate him, and finds their corroboration in facts as widely apart as possible, so the large group of monomaniacal visionaries convert everything analogous to their purpose, and derive fresh impulses from follies and delusions greater (if that be possible) than their own. Thus we find that homœopathy and hydropathy, like two drunken strollers, mutually support each other; and thus, too, mesmerism finds in modern necromancy, and in every delusion of sorcery and magic, current in all ages and amongst all peoples, the strongest proof of the verity of its doctrines.* We do not propose to treat these visionaries

* Magie Magnétique, ou Traité historique et pratique de fascinations, de miroirs cabalistiques, d'apports, de suspensions, de pactes, de talismans, de charme des vents, de convulsions, de possessions, d'envoûtements, de sortiléges, de magie de la parole, de correspondance sympathique, de nécromancie, &c. Par L. A. Cahagnet, auteur des Arcanes de la Vie Future Dévoilés, &c. Paris, 1854.

with contempt, or to assail their doctrines with abuse. The greater number deserve neither. The enthusiasm and moral courage the majority display, present, when considered abstractedly, something commendable. Some of them, at least, are *honest* (albeit ignorant and superstitious) seekers after truth; and are not to be confounded with the knaves who prey upon their simple credulity. Their good qualities, indeed, commend them to our sympathy; and being so commended, we think we shall do them service if we endeavour to trace to their origin the influences by which honest, pains-taking, and earnest inquirers into truth are led onward from one absurdity to another, each greater than the last, until the climax is reached, and monomania, insanity, or imbecility, tragically end the comedy.

What we find before us is just this. A very considerable number of persons write books, read them, and associate themselves together, whose object is the investigation of propositions and the promulgation of doctrines which either shock our common sense by their absurdity, or wound our religious feelings by their impiety, or excite our contempt by their inanity. The absurdity, impiety, and inanity are so flagrant, indeed, that it seems to be hardly necessary to state in so many words these characteristics of the doctrines advanced; but, on the other hand, the promulgators themselves entertain a widely different opinion of their views and of their proceedings. In their own estimation they are persecuted lights of the world, of whom the world is hardly worthy; martyrs to science and to progress; unappreciated and incomprehended investigators of the mysterious unknown in physics and psychology; discoverers of new worlds of phenomena and forces. Why this difference?

We believe the whole class of visionaries to be more or less affected with monomania. They have eaten of "the insane root which takes the reason prisoner." Not insane metaphorically, but practically and pathologically; they are inoculated with a "fixed idea" by a process of mental operations not dissimilar from those known under the term *electro-biological*, or are actually insane.

We will first describe the symptoms of the case. As they show themselves in the *mass*, the nature of the delusions of these monomaniacs is tolerably well shown by the titles of works published by M. Cahagnet, according to the list on the paper cover of the work before us. First, we have the delusive ideas entertained as to the relations of the soul with the present and future world, indicated by—

"*The Arcana of the Future Life Unveiled;* a work containing unanswerable proofs of the faculty possessed by magnetic somnambulists of seeing the departed, and conversing with them."

"*The Sanctuary of Spiritualism,* a study of the human soul and its relations to the universe, in reference to somnambulism and ecstasy; showing to every person the means of entering into the ecstatic state at will."

"*The Light of the Dead*, or studies, magnetic, philosophical, and spiritualistic; dedicated to the liberal thinkers of the nineteenth century."

"*Magnetic Spiritualistic Encyclopædia*, treating specially of psychological facts, magnetic magic, Swedenborgianism, necromancy, celestial magic," &c.

The readers of these works, with other human beings, are gregarious; they communicate to each other their ideas and discoveries—the means being a *society*, and an organ of the society, or *journal*. This is—

"*The Spiritualistic Magnetiser, a Journal* of the *Spiritualistic Magnetisers of Paris*, treating of very curious facts of apparitions, of possessions, of psychological questions."

The *material* forces, as distinguished from the spiritual, are not neglected by the sect of whom M. Cahagnet seems to be the apostle; for the "Od force" of Reichenbach is believed in and discussed for them by that writer.

"*Odic Magnetic Letters* of the Baron von Reichenbach, translated from the German, with an estimate, by the author of the '*Arcana*,' of the phenomena of the fluidic currents which the three kingdoms manifest."

It is always a weakness with these visionaries to believe that their doctrines possess a practical application; they therefore laboriously endeavour to demonstrate their usefulness. Hence books like the following:—

"*The Treatment of Disease* by the *Extatic*, Adèle Maginot. Studies on the medicinal properties of 150 of the most common and best known plants, with various methods of magnetization."

The "Magie Magnétique" is a work which is best described in the author's own words:—

"It is the fruit of patient studies, of patient observation, and not less patient experiments. It will teach little to the studious magnetiser, for it appears to be reserved for us in this age of darkness (termed enlightened) to have no other duty than to go back to the past, for the purpose of studying the labours of our fathers, and to appropriate them, as a proof of progress, and not as a simple heritage; and to forget them sufficiently, that we may loudly exclaim, 'In no age were discoveries so common as they are in ours.'"

These labours referred to is the farrago of superstitions and frauds, which M. Cahagnet catalogues on his title-page, and the facts of which he receives as philosophical facts worthy of all credence, and clearly proving the doctrines of magnetism. An alphabetical list of these labourers comprises the most anomalous names—Gregory VII. precedes Hermes, Isaac, Jambres, Jannes; Medea, Merlin, and Mesmer come together, and are followed by Swedenborg, Trois Echelles, and Tubal-Cain. Everything absurd unintelligible, and mysterious, is within the domain of magnetism. The following is a portion of M. Cahagnet's decalogue—a sort of catechism of the powers of the so-called science:

"Can the cataleptic condition be induced by magnetism?—Yes.

"Can the powers of a magnetic subject under your influence be diminished or increased threefold?—Yes.

"Can the effects of attraction be produced in this subject—as all magnetisers declare they have produced—not only on living beings, but on dead matter?—Yes.

"Can they cause the suspension of material bodies by the same effect of attraction?—Yes.

"Can certain subjects in the magnetic state perform certain gymnastic movements, which are contrary to the laws of anatomy?—Yes.

"Can a being in that state attain to a height far beyond that of his natural size?—Yes.

"Can he walk upon points of support contrary to the constitution of his nature and the laws of equilibrium?—Yes.

"Can he induce on his person excessive local and general inflammations?—Yes.

"In this state can he, with his eyes closed, see either by the nape of his neck, the plexus, the heels, at immeasurable distances, and hear what is said there?—Yes.

"Can the so-called spirit, when separated from matter, form material vehicles? (*des apports matériels.*)—Yes.

"Can the lucid speak many languages previously unknown to him, and acquire a knowledge of science of which he has always been ignorant?—Yes.

"Can he in this state defy the action of fire or of poisons?—Yes.

"Can he enter into communion with the dead, speak to them, and obtain useful knowledge from them?—Yes.

"Can the magnetiser beset his subject by sounds which he causes him to hear at a distance? In like manner, from a distance, can he exercise an attraction upon him? produce before him ghosts or other phantasms? and compel him to do things contrary to his peace of mind, to good morals, and to honour?—Yes.

"Can the magnetiser in this way render his victim idiotic and imbecile, or even destroy him, without leaving any visible marks?—Yes.

"Can he communicate to him any particular disease, or deprive him of the use of a limb? Yes.

"Can he give him blows at very great distances?—Yes.

"Can he bewilder him on his way, make him leap ditches (as you have assured me), place stumbling-blocks before him on the level road, cause robbers or ferocious beasts to appear to him?—Yes.

"Can man cast stones into distant localities without being seen, bewitch the soil, gardens, animals, and men, as is stated in all works on sorcery?—Yes.

"Can he act upon masses of men at the same time, causing them to see, touch, and eat things, real apparently, but in fact imaginary only?—Yes.

"Can man have disembodied spirits subject to him, and be served by them?—Yes.

"Can he at his pleasure excite, or cause to cease rain, winds, hail? —Yes."

The supposed questioner (for the book is in form of a dialogue) candidly remarks that assertion is not proof; whereupon the author declares that although he cannot show the means by which all these marvels have been effected, he will prove that they have been done, and that it is possible to effect them "by magnetism as the principal agent, aided by the infinite *combinations* of the human mind." The proofs consist in a number of stories drawn from every branch of the literature which treats of the wonderful and mysterious, and details of experimental researches; they consist principally of three kinds—firstly, absolute inventions; secondly, tricks of conjuring and swindling; thirdly, phenomena analogous to those of mesmerism and electro-biology.

We shall defer for the present a more detailed account of the contents of this curious pathological work, reserving to a later period special instances of monomaniacal delusion which it contains. In the meanwhile we will adduce illustrations of this kind from other and very different sources, analysing in particular some of the monomanias with which the English public is perfectly familiar. The trivial and absurd are just as important by way of illustration as the more pretentious; we shall, therefore, select instances of both kinds. The better to display their source and progress, which are closely connected with *the association of ideas*, we shall commence with those in which a given idea is predominant, and the idea we choose is the *religious*.

The religious element of man's nature has (we need hardly remark) a close connexion with the occult and the wonderful. "*Faith is the evidence of things unseen*," of things beyond reason and observation. M. Cahagnet shows that unhesitating faith is the basis of all his occult knowledge. The whole power of "magnetic magic" depends on the will; but, he adds, the human will has never been defined. "Faith alone," he remarks, "is the *soul* of the will; it is its principal agent, its lever, its power, its life." In the condition of absolute unhesitating faith "there are no longer impossibilities; the ordinary laws of nature are abrogated, and the inexplicable is revealed by the incomprehensible." It is a natural consequence of this state of mind that they who have faith in the occult in philosophy are not slow to add to it the mystical in religion. Whether there be a phrenological reason for the connexion between religious delusions and more material follies in the contiguity of organs, we do not pretend to say; certain it is that they are often intimately allied in all these various forms of monomania. As illustrations we subjoin the following. Morison, the *pill-merchant*, like many other fanatics, believed in his own absurdities. He maintained that the cause of disease in man which purging cleanses away, first entered into the blood of Adam and Eve at the fall, and is the infection, *par excellence*, of man's nature. He certainly, if the history of his own case be credible, was a confirmed hypochondriac. He says:—

"Reader, this was the cause from the beginning of my disease—want of all rest and comfort, and loss of fortune. *I frequently thought that I should go mad, and that I was possessed of a devil within me.* In the first periods of it, and when my other feelings were still acute, I would have taken up my abode in the sandy deserts of Africa to obtain a few nights' sound sleep, the common solace of mankind. You cannot imagine to yourself the anguish and pain of it; yet no one knew how to give me relief. At its commencement, thirty-eight years ago, it was only a simple humour that settled there. . . . . . Reader, all your diseases and pains arise from a like cause; they must proceed from a humour. I defy all ingenuity to establish any other cause."

Now, a monomaniacal idea of this kind is not of itself incompatible with shrewdness and cunning. Numerous instances of successful religious fanatics, the founders and leaders of sects, might be adduced in proof. Even where the ideas themselves are incoherent and the practical results ridiculous, the monomaniac exercises, in virtue of his singleness of idea, a power over weak and ignorant minds. There is a certain empiric wanderer about town, who is perhaps better known from his propensity to disturb public meetings by his wild crotchets than by his "Asthmatic Lamp, or Air Flame Magnet to Breath.—(xii. 5; Job. xxvii. 3)." The reader who is familiar with the literature of the insane cannot fail to recognise a well-known style in the following literal extract from this monomaniac's description of his lamp:—

"The *inventor's* reasons for a *dry air fumigation*—night and morning—in scrofula, in *cancer*, and in all cutaneous *eruptions*, is based on the *deliverance* from 'king's evil,' *by its drying up after eighteen day's use*, in the child '*Owen*,' of No. 7, Derby-court, Jermyn-street, which event to *one* 'that is ready to slip with *his* feet,' is in perfect keeping with the *Almighty's* laws, viz. that as the flow of tide twice in twentyfour hours purifies the waters of the globe, and in its undulatory movement, acts on the local temperature by atmospheric pressure, so in like manner under analogy with, and IN, the use of 'sulphate of zinc' in drinks, as giving a *vitriolic* cause for *morbid action*, or sudden clotting of the blood in persons."

The author of this insane rigmarole is only not a dangerous fanatic because he has not even an elementary knowledge of any science, and cannot write two ideas in normal association; yet even he has his followers and votaries. It is not long since, when an explosion of a coal mine in Scotland took place, that this monomaniac hastened to the locality, persuaded the friends of some miners that were hurt to submit them to his treatment; and when—as a necessary result of his foolish interference—he was put upon his trial for manslaughter, and a verdict of not guilty was announced, an enthusiastic audience of his admirers could not repress their delight at the result!

The heterogeneous sect which Hahnemann founded presents an interesting illustration of the working out of monomaniacal ideas from a common root. It has been long known that active medicinal agents produce morbid conditions not unlike those which they cure or relieve. Thus, a purgative (an irritant to the mucous surface of the intestinal canal) will relieve a disease (diarrhœa) caused by an irritant. An inflammation artificially excited on the skin checks inflammatory action elsewhere. The entire group of remedies termed *counter-irritants* may be mentioned as belonging to this class. Now, a German physician, following out this idea, found it applicable under circumstances not generally recognised or known; and passing to that hasty generalization from a few instances, which is the leading fault of all investigation of the occult, he promulgated the fundamental doctrine expressed briefly in the phrase, *similia similibus curantur*. This doctrine would soon have passed into the limbo of those hasty generalizations which specially abound in the history of medicine, leaving behind it a useful residuum of facts, had not Hahnemann been infected with that monomaniacal temper which exercises so potent a control over weak and ignorant people. Under another form, namely, the doctrine of *signatures*, the primary idea had already had its day, but Hahnemann claimed a special revelation to himself of the healing power of nature. In the doctrine of signatures it was maintained that the Divine mind revealed them to all alike who could read the signs or signatures in the external appearance of plants. "Eyebright," to the sagacious observer, revealed its valuable properties in the cure of dimness of vision by the form of its flower, and "liver-wort" its valuable properties in hepatic diseases by its liver-like appearance. Hahnemann, cunningly wise (as monomaniacs can be)

pretended to found his views both upon experience and revelation. He remarks, as to his Divine mission:—

"It was high time for the wise and benevolent Creator and Preserver of mankind to put a stop to this abomination (the ordinary method of treating the sick), to command a cessation of those tortures, and to reveal a healing art the very opposite of this, which should not waste the vital juices and powers by emetics, perennial scouring out of the bowels, diaphoretics, salivation, &c. . . . . nor, in short, instead of lending the patient aid, to guide him in the way to death, as is done by the merciless routine practitioner. . . . . it was high time that He should permit this discovery of Homœopathy. By observation and reflection I discovered that, in opposition to the old allopathic method, the true, the proper, the best mode of treatment is contained in the maxim," &c.

Amongst the delusions of the insane hypochondriac none is more usual (as is well-known to practical men) than that the health is suffering from some constitutional taint or disease. We have seen that Morison entertained this delusion. In this country the syphilitic poison is most usually fixed upon by the insane as the imaginary *fons et origo mali.* Now, in Germany there is a very generally prevalent theory that *scabies* is a constitutional disorder, leaving behind it important changes analogous to those caused by the syphilitic virus. Hahnemann's monomaniacal mind naturally seized upon this idea, and incorporated it with his other doctrines, thus giving rise to his psora doctrine. Psora (or itch) is, according to his views, "the origin of at least seven-eighths of all chronic diseases," the other eighth resulting from "syphilis," or "sycosis," or a union of either or both with psora. It is, he says, a sin against humanity to consider the itch eruption as a local disease, and treat it by ointments or washes to drive it off the skin.*

Hahnemann invoked experience by experimental researches into the action of remedies, or "provings," in the jargon of the sect. The basis of the method is, that a person who "proves" must take fasting about the same dose as is usually given in diseases, he must remain some hours longer fasting, and he must carefully observe himself.† A very slight knowledge of the physiology of the nervous system is only needed to convince the reader that no better method could be devised for exciting those sensorial changes which unquestioning faith and morbidly excited acts of *attention* are well known to produce. Hahnemann very quickly, therefore, came to the conclusion that very small, indeed immeasurably small doses of remedies produced powerful effects.

* The British Journal of Homœopathy, July, 1849, p. 349.
† Ibid. p. 341.

From this to the doctrine that mystic forces developed by trituration and succussion rendered them more powerful, or "potentized" them, was only a step, and so the infinitesimal theory was founded. We have, then, in Hahnemann's writings these notions developed :—1. That he was specially inspired by the Divine mind, and that therefore the system he proposes to overthrow is utterly wrong. 2. That Divine Providence has provided special medicinal agents antagonistic to every ill. 3. That there is an occult source of bodily disorder from which every special disease arises—psora. 4. That there are occult powers in the means provided by Divine Providence, which may be developed by certain manipulations, namely, trituration and succussion.

These insane propositions (for no one of which there exists the slightest foundation in fact) were not so insane as those to which they have given rise in minds less disciplined and cultured. We have already remarked how inevitable is the tendency in persons of this monomaniacal mental character to develop their notions, and draw within their circle any morbid analogous absurdities. The published discourse of the Reverend Thomas Everest, Rector of Wickwar, preached in London, in aid of the Hahnemannic Hospital, presents us with an instance in point. This divine, who boasts of "some years of intimacy" with Hahnemann, conceives that he has discovered the doctrines of his friend in the Holy Scriptures. Here we can trace the operation of the associating faculty :—

"At the fall of man, sin entered into the soul, and disorder into the physical frame (with which that soul is connected); at the same moment, God sent his Son to repair the mischief, and He bade his ministers preach the Gospel and heal the sick; that is, cure the moral disorder and the physical disorder together; and for nineteen hundred years that precious wisdom has cried in the streets unheard."

There is therefore, according to both Morison and Everest, an original physical taint as well as a moral taint derived from the fall. In the command of our Saviour to his disciples—"Cleanse the leper"—Mr. Everest finds grounds for believing the leprosy of the New Testament and the psora of Hahnemann to be identical. He conceives, also, that this *corporeal* taint is also the source of sin; or, in his own words, "irreligion is the daughter of internal disorder." Now, as all internal disorders depend on psora, it follows that an anti-psoric homœopathic treatment is an excellent adjuvant to spiritual purgation. Mr. Everest therefore very logically (your monomaniacs are unflinching logicians) recommends that a continuous medicinal treatment, founded on these principles, be begun in childhood, with the hope thereby "to anticipate disorders, to restore harmony, to combat the internal *psoric*

tendencies, and to procure a patient hearing and kindly reception of spiritual ministrations." Here is the fundamental idea of Morison, but developed in a more theological manner, by an association with ideas already grafted in the mind of the reverend divine.

This religious element of homœopathy has assumed a more eccentric development than even this of Mr. Everest's. A French writer speaks of Hahnemann as "the new Evangelist," and "the most inspired of revealers." The "school" of Rio Janeiro, from whence this notion has arisen, is an excellent instance of the more developed forms of the Hahnemannic monomania. The founder of it adopted the idea of potentization, and invented machines for succussing or shaking remedies, and thereby increasing their medicinal virtues. It would be wearisome to the reader to specify the follies of this sect in this respect. This school also extended the religious branch of the delusion, and drew a parallel between Hahnemannism and Christianity. Prince Alphonso, heir apparent to the throne of Brazil, died in a few days after his birth, for want of homœopathic treatment. A European promulgator of the views of this school (author of "Doctrine de l'Ecole de Rio," &c.) maintained that for medicine to become, like other sciences, properly Christian, it was necessary that a victim should be offered up as an expiatory sacrifice, "to conquer the indifference of the vulgar." This expiatory victim "for the physical redemption of humanity" was this infant prince. "It seems," he concludes, "that it is only by an excess of evil that man can return good. In order that humanity should renounce the worship of false gods, nothing less than a DEICIDE (*sic in orig.*) was requisite. It is by a REGICIDE (*sic*) that allopathy behoved to mark its last hour."

We will follow this monomaniacal association of ideas a little further. Out of the homœopathic hallucinations the *isopathic* arose. According to isopathy, all contagious diseases contain in their contagious matter the instrument of their cure. This was the notion of a German veterinary surgeon, named Lux,—arising, manifestly, from the Hahnemannic dogma. All contagious diseases were therefore recommended to be "potentized" into remedial agents—variolitic matter for variola, the itch-virus, or "*psorine*," for the itch, &c. This latter became a great remedy for all the so-called psoric diseases (nine-tenths of all diseases whatever). Things horribly filthy or ludicrously nasty, were, on the isopathic principle, gravely recommended to be "potentized" and administered. A bug in the thirtieth dilution (the decillionth), cured the inflammation arising from a bug-bite. But we will not record the filthinesses of these monomaniacs; suffice it to say, that *no animal product whatever* is too abominable to be "potentized" and administered as an infallible remedy!

Amongst the more curious divergencies of the homœopathic ideas is that towards mesmerism, the point of junction of the two being in the monomaniacal idea of the "Od force." We have already seen that, according to Hahnemann, certain occult forces can be developed in matter by trituration and succussion; well, a Mr. Rutter invented a "magnetoscope," whereby magnetic currents could be detected passing through the human body. The instrument was analogous to the "odometer" of the late Mr. Mayo,—the same in principle, a little more complex and scientific-looking in construction. Great was the astonishment and delight of Dr. Quin and other leading homœopaths to find that when their globular nonentities were tried by it, by being placed in the hand of the experimenter, they proved to have a most powerful magnetic influence—not less powerful, indeed, than Reichenbach's favourite "rock-crystal"! Mr. Rutter was almost worshipped, as may be gathered from the following extract from a lecture on these experiments by Dr. Quin:—

"I feel confident that you will agree with me, that science has made a gigantic stride by the philosophical instrument and important discovery of that gentleman, and that homœopathic practitioners especially are greatly indebted to him for having proved the physical action of our remedies, in infinitesimal quantities upon the human body. . . . . The only reason for sorrow is, that our revered master, Hahnemann, is not alive to witness this triumphant proof of his own great discoveries," &c.

Dr. Madden soon showed the true nature of this delusion. He found that any *anticipated* motion of the magnetoscope took place invariably; in short, that the movements depended upon the *unconscious* but suggested muscular movements of the operator; and so the bubble burst.

The study of the occult in modern times is no more profound than in the more ancient. The potentization of medicinal agents is little different from the alchemical notions of an elixir vitæ; delusions as to the divining rod, and the various modifications of the "Od force," may be compared with the mineralogical researches of the alchemical philosophers. Even the metallic rings and other electro-biologic and mesmeric agents have their parallels in ancient times. The Æs Cyprianeum of Alexander Trallianus is the exact analogue of nickel in the hands of a too-celebrated English mesmerist. The subjoined remedy for the colic is as likely to be beneficial as a magnetic ring, or a "potentized" particle of matter:—

"Annulum ferreum accipito, ac in circulum ipsius octangulum efficito, atque ita in octangulum inscribito φευγε, φευγε ιου χολη ἡ κοροδαλος ἐξητἐι; hoc est, Fuge, fuge, heu! bilis alauda quærebat subjectam autem figuratam in annuli caput scribito ℵ. Hujus, magnam habui experientiam."

An iron ring (made, we ought to add, on the 21st day of the moon) is the "base" in the above prescription. "Take an iron ring, make in its circle an octangle, and thus write within that octangle: 'Fly, fly, alas! bile the lark excites.' But inscribe the subjoined figure on the head of the ring: ℵ. I have had great experience of this."

Shade of Hahnemann! thy predecessor, Alexander, was thy equal not only in "proving," for he also rivalled the filthiest of thy isopathic followers in the nastiness of his remedies.

In the preceding illustrations we have confined our attention to occult force, used for *good* ends, or, at least, for things that may be honestly desired—as health, riches, and the like. Man has, however, been involved, from time to time, in delusions as to occult EVIL powers. These are, indeed, much more frequently the subject of monomaniacal delusions in the insane than of popular aberrations. The terrible times of witch-finding and witch-killing are full of horrifying proofs to what an extent delusions like these may prevail. The ideas as to the nature of these EVIL forces differ according to the *associated* ideas—*i. e.*, the educational prejudices and pre-conceived ideas of their victims. Amongst the Slavonic peoples, the horrible superstition termed *vampirism* seems to have been current, like witchcraft amongst the Celtic and Teutonic. So late as the beginning of the last century, it was epidemic in Bohemia, Moravia, Silesia, Poland, Hungary, and Wallachia: there was hardly a village that was not said to be haunted by one of those blood-sucking demons termed vampires. Our Gothic and Teutonic ancestors believed in some similar delusive appearance, and evil agents, termed Stryga, or Stryx. We shall see shortly that a being, with *bat*-like wings, haunted M. Cahagnet's midnight hours; vampyrism, nevertheless, is not (strange to say) amongst his marvels, although we have a conviction it has not escaped his bibliomaniacal researches into "the black art." It is not uncommon, since magnetism and electricity have been made popular sciences, to meet with monomaniacs who have a fixed idea that they are secretly galvanized or electrified by a distant and inveterate foe. Even yet the monomaniacal notion of active sorcery and witchcraft may be met with in practice, and a poverty-stricken decrepit woman becomes an object for the hate and dread of the miserable hypochondriac. The author of "Magie Magnétique" expresses his entire belief in the various tales of sorcery with which mediæval literature abounds—*e.g.*, he quotes, approvingly, the story from Boëthius' "Annals of Scotland" as to a certain King Duffus (?) of that country, who was bewitched by certain sorcerers by means of a waxen image of him, which they *roasted* (*sic*) before a little fire; and who was only restored to health by the discovery and combustion of the malefactors. He quotes, also, a story of seventy sorcerers who were put to death, in 1670, in Sweden, who (amongst other crimes) destroyed those that displeased them by striking the air with a wooden knife. Some hundreds, indeed, of such sorcerers were burnt in that country, without any apparent diminution of their numbers. But M. Cahagnet only mentions these facts historically; he relies upon very recent instances for his proofs, of which the following is an example. It is taken from an author* whom he describes as one who appears to him to be a very learned man, full of good faith, and above all suspicion of interested motives or of weakness of mind:—

"It was in the month of March that three itinerant magnetiques, paid by I know not whom, began in the darkness, and at a distance, to magnetize me, and to develop phenomena which I could not explain, and which occupied much my attention. I heard persons abusing me, but I could not distinguish them; I had headaches, was restless, the nervous system began to be in an abnormally irritable state, &c. Subsequently, tormented by the voices which menaced and insulted me, *especially during the night*, and believing that the family Lavaud betrayed me, for their voices were imitated for the purpose of deceiving me, I left the residence where I had been loaded with kindness. . . . . Some time after this change of abode, the three wretches who had rendered me so unhappy, and who had calculated beforehand the advantage of isolating me, took all possible means for bringing me wholly under their influence, and succeeded towards the end of May. At that time, one evening, at the moment between waking and sleeping, when my will and powers of resistance had left me for a moment, I was magnetized in floods, if I may so express myself; and in the morning I was wholly at the mercy of my persecutors,—or, in other words, three strangers, whom I had never seen, did, unknown to me, and in spite of me, so take possession of both my moral and corporeal freedom, that they saw by my eyes, heard by my ears, touched by my hands, . . . . &c. I pass over in silence" (continues M. Cahagnet) "the thousand and one miseries this gentleman suffered; he laid an information before the tribunals, and caused a petition to be presented to the chamber of deputies, through M. Croissant, with the view of directing the attention of the government to the occult machinations of magnetism. That petition was not taken into consideration. To support the statement as to his being bewitched, he quoted the case observed by Dr. Recamier, member of the Royal Academy of Medicine of Paris. This recent fact I subjoin."

The case there subjoined by M. Cahagnet is that of a peasant who at the same hour every night, heard a deafening noise, as of a caldron

* Observations de Magnétisme Occulte, par Emile Roy, docteur-médecin, ancien chirurgien-major, p. 1840.

or iron pot struck very powerfully; it prevented him sleeping, and he thought that he had either an affection of the brain, or some disease of the organ of hearing. He therefore consulted Recamier, who, after making the necessary inquiries, ascertained that a blacksmith, who lived in a village at some distance from the patient, had a spite against him for some trifling offence or other, and with a view to annoy him, struck an iron pot every night at the same hour; and although no one else, not under the influence of the smith, could hear the noise, the unhappy peasant heard it as clearly as if it were in his room. Recamier took the necessary steps to put a stop to the smith's spiteful hammering, by frightening him with consequences, and the magnetized was from that time let alone.

Amongst other similar stories related by M. Cahagnet, is one he quotes from the *Presse*, from which we learn that Pascal had a confirmed belief in the power of good and bad spirits, for the following written amulet was found in a fold of his doublet, wrapped up in parchment:—

"L'an de grâce 1655.

"Lundi, 29 Novembre, jour de Saint Clément, pape et martyr, et autres du martyrologe.

"Veille de Saint Chrisogone, martyr, et autres. Depuis environ deux heures et demie du soir, jusqu'à environ minuit et demi.

"Feu.

"Dieu d'Abraham, Dieu d'Isaac, Dieu de Jacob.

"Non des philosophes et des savants.

"Certitude, certitude, sentiment, joie, paix.

"Oubli du monde, et de tout, hormis Dieu.

"Joie, joie, joie, pleurs de joie."

This amulet of Pascal is described by M. Lélut, member of the Institute, and principal physician to the Asylum Salpétrière. Pascal (we are told) was bewitched before he was a year old, by an old sorcerer who received alms at his father's gate, and who confessed to having cast a spell over the child. The old fellow sacrificed a cat to the devil to break the spell, but without success; he then made a poultice of various herbs, which he had got a little girl, aged seven years, to gather, and applied it to the abdomen; the child fell into a lethargy, but returned to life at midnight, as the sorcerer had predicted.

The true character of M. Cahagnet's mind — namely, reasoning mania—may be gathered easily from the details of a successful *experiment in incantation* made by him. As it is calculated to throw light, not only upon the state of mind of this mystical lunatic, but also upon these maniacal delusions generally, we subjoin it. M. Cahagnet introduces his story by an explanation of the way he was led to make his experiments. He says—

"M. Renard, of whom I have spoken to thee, is my master in all these studies. He is possessor of an excellent library, as I have frequently observed, in which he often left me to rummage out and read at my leisure all the conjuring books therein. When I had got thoroughly crammed with their gibberish, I remarked to M. Renard that it was of little use to read only these old volumes, and stop there. I wanted specially to know whether these magicians, sorcerers, cabalists, astrologers, &c., had said anything true, or were only pur-blind. Would you, on some fine midnight, make a regular conjury with me in the midst of the forest of Rambouillet, in the most detestable and diabolical locality possible? I should like to see the devil face to face, and have a pull at his beard, &c. My friend frowned, compressed his lips, and assumed the most picturesque figure of a sorcerer I had ever seen. I thought for a moment that he was the devil himself. He gave me no answer. 'Oh! indeed!' I said, 'are you afraid—you the terror of the country, before whom the children cross themselves, and the women tremble?' 'Yes,' my friend replied, in a tone which made me burst out laughing. 'Do not laugh, Alphonso,' M. Renard replied, 'and never you do that which you have proposed that we two do. I have made the trial once, and I never will repeat it.' 'What have you seen, then?' I replied. 'That which I not wish to see,' my friend answered. 'Let us cease this conversation, or change the subject. Only you remember my advice, and profit by it,' were the last words of M. Renard."

Now, M. Alphonso Cahagnet's curiosity was only whetted by this mysterious counsel. He "had never known what fear was, and he had so often escaped certain death that he had come to consider himself invincible." He considered the matter well, and asked himself what need was there to go into the depth of a forest and make a diabolical incantation when according to the Christians (*sic*) we had always near to us a good angel and a devil, and, according to the clairvoyants, a good and bad director? Why not call these directors up? This conclusion appeared sound; so deciding that night was the best time, he wrote on a piece of paper, and signed the following exorcism:—

"Au nom de Dieu tout-puissant, ton créateur et le mien, je te prie, ange commis à ma garde, de m'apparaître cette nuit, afin de me prouver la réalité de ton existence."

Or in other words he summoned his good angel in the name of God, their common Creator, to appear to him, and prove thereby his existence. With this paper under his ear, and nothing doubting as to the result, M. Cahagnet retired to repose. The result we will give in his own words:—

"It was in February 1841 that I tried this kind of communication. The first night I neither saw nor felt anything extraordinary; the second offered to me a phenomenon which astonished me enough not to desire to experience it again. I had only been in bed a few minutes. I was not asleep; at least I was certain I was not, when I felt my left arm gently drawn out of bed; then a stronger force drew upon my leg, so that my body followed my two limbs, and I fell upon the floor. I immediately exclaimed, 'Oh, my God! what is it that this means?' I had scarcely uttered than I replaced myself in bed, with the complete consciousness that all that had passed was only the effect of the imagination. I began to reflect upon what I had done, and upon the consequences which might follow upon my exorcism. I did not like to withdraw from the attempt, so I continued to place my little bit of paper under my ear every night. I was quiet for some days, and I already doubted the result of my experiment, when I one night begged a good aunt of mine, who had been dead some years, to appear to me instead of my guide. This aunt loved me much when on earth; she had tried by every possible means to induce me to observe what she termed my *religious duties* without success. If I had thought I should meet in the churches no souls but those as pure and angelic as her own I would have gone every day to solace my wounded spirit within their kindly sphere, but the intolerance of the priests had made me an atheist. I conjured, then, this noble creature to appear to me, if she were able to do so. I was astonished to be awoke on the same night by the sound of a very powerful bell, the clapper of which struck three times, and three strokes each time. Hardly fully awake, I saw before me the son of my aunt, my cousin-german, who had died after her. I entered into conversation with him, and was really quite astonished to hear him speak to me of the spiritual world, of the goodness of God, and of the need of pure religion. When this cousin was on earth he thought as I did; indeed, I think I owed a little of my scepticism to him. I replied that I was better disposed towards these matters than when he was on earth, seeing that I read books which had enlightened me, and that at that moment I was endeavouring to enter into communication with spirits so as to assure myself of their existence and of a future life. I added that I had called my aunt for this identical purpose. I had not finished the last sentence when I saw that good aunt standing at the foot of my bed, in an attitude the most majestic possible, stretching one arm toward heaven, pointing me to it with her finger, and saying to me in a voice inexpressibly tender and touching:—

"'Alas! Alphonso, do you still doubt the power of God?'

"Overwhelmed by these words, I endeavoured to revive my scepticism by a few unconnected expressions. All disappeared. I lighted a candle to see what o'clock it was. It had just struck four. Some days now elapsed without anything happening. Another night I heard the sound of that same bell striking the same number of times. I opened my eyes, and I saw a human head hover over my bed, the most hideous imaginable; it was supported by two large bat wings; its eyes were of fire, and seemed to pierce me to the heart. I was so enraged to see such a monstrous creature that I repelled it with voice and gesture, sitting up in bed the better to defend myself from contact with it. Not being able to succeed, I invoked my good angel and my aunt. Hardly had I expressed the wish than all disappeared. On the night following the same noise again awoke me, and I saw kneeling before my bed a woman whose long black hair concealed her face, but whose wicked intentions I knew, and which she avowed to me without my knowing the motive. I was obliged to have recourse to the same invocation to rid myself of this infernal woman. I began to write of these visions, and noted the hour at which each occurred. Similar noises and visions, more or less fantastic, continued for some months. One night I had hardly laid my head on my pillow when I felt it to move so as to raise me up at least six inches. I thought this was in consequence of the breathing of a strange head beneath mine. I asked (I know not why), 'Is it thee, my good director, who art here?' 'YES, YES, YES,' a voice answered at three distinct intervals, speaking from just below my pillow. I was terrified by the answer, and asked no more that night.

"The next day the noise changed; it was not the sound of a bell which awoke me, but the same blows struck in the same manner with rods, which I thought of iron from the clear sound they made; the same movement was felt under my head; I again asked if it was my director, and it made me the same answer in the same way. Less afraid than the night before, I said to him, 'If thou be my good angel, thou hast a name; if thou comest to me with a good intent, it is right that I know how to address thee when I have need of thee—tell me then thy name. The word AGOOR was pronounced thrice with such a prolonged detonation of sulphur that I beseeched God never to let me hear that name again."

In this way M. Cahagnet goes on describing his nocturnal spectral illusions. For three years, indeed, he tells us he heard voices of every kind; saw all sorts of visions; felt various sensations; and especially had numerous previsions. He could not get rid of them, and obtained only a temporary relief by calling upon God for help. He applied to his master, M. Renard; he read medical works on various diseases, but in vain. The first clairvoyante, however, whom he consulted told him that he was under a spell. This self-inflicted suffering, so madly misunderstood, had an evil effect on others as well as on himself. With great naïveté he further relates how an inhabitant of Troyes, a reader of his *Arcana of the Future Life*, came express to Paris to state his position. He and two others having read the "Occult Philosophy" of Agrippa, determined to adopt the methods of incantation detailed in that work. They made the requisite arrangements, reached the place appointed, traced the circle, and commenced the ceremony, and were forthwith assailed by a shower of stones, groans, and by hisses, doubtless by hidden spectators. Nor was this all. One of the three had lost 60,000 francs by his foolishness, and was reduced to poverty; another lost an office under government which he held; and a third so mismanaged his affairs that he was sent to prison. These sufferings were all, however, simply proofs of their truth and sincerity! Compacts with spirits are as fully established (M. Cahagnet remarks) as anything in the world.

Those who have read that sad chapter in European history which unfolds the treatment of WITCHES and WARLOCKS, cannot fail to recognise in this M. Cahagnet one of those men who would have victimized hundreds by his follies, and would himself have finally been the victim of the superstition, ignorance, and credulity of society. Reading these details, so deliberately and calmly given, one can hardly believe that it is now the middle of the nineteenth century. And as formerly the deluded creatures named their familiar spirits Pyewacket, Vinegar Tom, Grizell Greedygut, Swein, &c., so do these visionaries name theirs. One Colonel Roger is quoted, who formed a cabalistic club of nine members, whose object was the practice of the black art, and who had a certain spirit, termed Mikenas, at their call. Mikenas would fetch and carry like an errand-boy.

> "*Prospero.* What, Ariel; my industrious servant Ariel?
> *Ariel.* What would my potent master? Here I am."

Mercurial water is wanted, Mikenas is invoked, and the flask is seen to place itself of its own accord on the window-sill. The greater number of the members took snuff, and complaining of the bad quality of what they had, desired "narquitoche," a *recherché* American snuff not sold in France. A certain *lucide*, already known in London, we believe, to be an impostor, advised that money should be put down for Mikenas to get the much desired snuff. No sooner said than done. Mikenas was invoked—away went the money. Hey, presto! in a minute came the snuff! The following story of the colonel's of their cabalistic doings is too curious to be omitted:—

"Know that the members of this club have, in their cabalistic articles of faith, come to the conclusion that it is possible for elementary [spiritual] beings to be transformed into material beings, as thou may read in a book entitled, 'Le Comte de Cabalis,' &c. I have already referred to this subject in our conversation on cabalistic mirrors, when explaining that the cabalists admit that the elements are only composed of such beings as ourselves, but without our immortality, which they do not possess and cannot, except by being united to the sons of heaven, who are our *seigneuries*. The chief of the colonel's club (following the advice of the clairvoyante who enlightened them) had concluded a spiritual marriage with a sylph, a spirit of the air; but to the end that this spirit should be visible and palpable to his material senses it was necessary, naturally, that it should be materialized, and this could only be effected by the spirit absorbing those of our material substances the most adapted to its nature. This is what happened for six months. Every day this invisible spirit placed itself at table before M. Pic——, and devoured all the food which it had demanded from their clairvoyante, and which were expressly prepared for it. There only wanted a few months' more of preparation for this spirit to be rendered visible to the eyes of her *fiancé*. . . . . But the revolution of July came to dash this sweet hope by carrying off M. Pic——, who died some time after, and himself joined his *fiancée* in the spiritual world. What is certain in this account is this, that the material food placed every day at each meal on the plate of this spirit disappeared every time in the presence of those whom monsieur invited to dine with him, and this continued for six months. I have this statement from two eye-witnesses, Colonel Roger and M. Bodes, an octogenarian now residing in a *maison de vieillards*, rue des Recollets, in Paris."

The imaginary "G." of the dialogue exclaims, on hearing this recital, "Oh, my good friend, where are you leading me?"—and the answer boldly is, "To the search after truth"!

"O, Setebos, these be brave spirits, indeed!"

We cannot omit stating that M. Cahagnet claims the power of *charming away the clouds*,—relating numerous examples of his success, indeed, and giving names of witnesses and dates. We will quote an instance or two, for the delusion is novel, and is a useful illustration of pathological psychology. We subjoin the history of his first experiment:—

"I had just completed the perusal of M. Ricard's 'Cours de Magnétisme,' in which that author appears much disposed to admit the magnetic influence of man on the atmosphere. I was interested by this idea, and on several occasions I made experiments, which appeared to me to be tolerably successful. I communicated these attempts to M. Renard, who lives in the woods six hours out of twelve every day. This studious magnetist also made the same experiments, with the same success. He believed it was not possible to doubt this magnetic power of man over the atmosphere; but the question is apparently so ridiculous and nonsensical, as thou hast remarked, that we abandoned this sort of experiments. They were of this kind: when I felt a VERY ARDENT DESIRE to experiment, I went into a little garden that I have; I there collected myself for a moment, looking at the sky and at the clouds with which it was more or less covered. My imagination (or my *will*, if you like) was then excited, and I stretched forth my hands towards some cloud which I wished to stop in its course. After some minutes of this action and of this conviction, it seemed to me that the cloud took the direction which I had imposed upon it. I say, *it seemed*, because I DARE NOT SAY MORE. It was not once only, but MANY TIMES. I had not the feeling that the cloud was so distant as it really was. I likewise thought it to be of a *compact* nature, capable of resisting any pressure, and I then used a certain amount of force to push it, just as I would with a heavy weight."

M. Cahagnet also made the rain to cease, and the sky to clear up. The hyperæsthetic sensation described in the following (marked by us in italics) is very significant and characteristic, pathologically. Something almost identical with it has been described by M. Cahagnet before:—

"One day, when the sky was overcast, and a very small but heavy rain was falling, I went into my garden, animated with the conviction that I was about to disperse the clouds and procure fine weather. I commenced proceedings, and *passed into such a state that it appeared to me as if the bone of my head was raised some inches.* I was not long in perceiving that a beautiful blue crown formed above me, which went on gently enlarging until, in about an hour, the rain ceased and the sky was magnificent. Was it an hallucination? It might be; but on entering the house I got strangers to touch my clothes," &c. &c.

The clothes were *dry*, and therefore, &c. Subsequently, M. Cahagnet made some experiments equally brilliant and more satisfactory, under various circumstances, and in the presence of different witnesses—namely, M. Lecocq, of Argenteuil, watchmaker; M. Chevillard Médar, farmer; M. Gérard, cooper; MM. Lejeune, Ravet, Emmanuel, &c. Médar and Gérard call upon him to witness his exploits:—

"I said to Médar, I don't feel exactly in a condition for trying an experiment of the kind just now, especially on such large clouds; however, if you will both help me, we will try. Oh, said they, we will willingly. Then, said I, I attack the head of that one which is upon the other and dissolve it away;—and I stave in its belly, said Médar, with that sort of faith in magnetic facts which is daily exhibited. M. Gérard added, 'and I will take the tail.' We all three set to work. Seeing us thus engaged, we might have been compared with the three Horatii, setting aside the object in view. In ten minutes our cloud had gone to rejoin its companions in that vast ethereal laboratory which contains us, and was no longer visible to our eyes. M. Gérard said, It is true the cloud is gone, but has not the cloud below it absorbed it? Possibly, I answered; let us therefore set to work to open the belly of that one, and recover our cloud—what say you? We will do it if it be possible, they answered, but it is sharp work. Let us try, then, said I; and at the same moment we went at the giant with such force and energy, that it disappeared, like its companions, in about ten minutes. Imagine the enthusiasm and astonishment of my visitors, who from that day have continued to make experiments more and more demonstrative and conclusive."

Here end our illustrations. Well may it be said, by the men and women who pass their lives in the pursuit of delusive objects like those we have discussed—

——"we are such stuff

As dreams are made of."

We now propose to draw the moral deducible from these follies and crimes. The whole history of these varied delusions is a most pregnant chapter in the history of human nature. It unfolds to the thoughtful psychologist wonderful and important glimpses into the more hidden workings of the mind. What strange ground for belief!—what irrepressible convictions!—what trusting faith!—what horrible martyrdoms!—what cowardly fears!—what constant fortitude! And all based upon nothing more substantial than the fantasies of the imagination, or the knaveries of rogues! It is now well known that if the attention be strongly directed to any object by individuals predisposed to cerebral disorder, a condition of the cerebrum is excited closely allied to notional insanity, or at least to that state which gives rise to various disorders of the nervous system. If the directing cause be something external to the mind, the phenomena induced come under the categories of the mesmeric, biological, &c., and frequently give rise to the erroneous deduction that occult material forces present in the external directing agent are the cause of the phenomena themselves. The attention may, however, be excited to special action by the *internal* operations of the mind itself. When this happens, the representative faculty acts directly upon the cerebrum, induces corresponding change therein and these return, as it were, to the mind, in the form of spectral sensations, perceptions, imaginations, and associations of ideas,—not, however, easily recognised as morbid, when the mind is enfeebled, or the reasoning powers imperfectly developed. It is this morbid mental and cerebral condition which is most usually present in enthusiasts and visionaries, in whom the attention has been strongly fixed on a special object of thought or perception, in consequence of a predisposing love of the marvellous, or from the compelling force of the circumstances in which the individuals are placed.

In considering these forms of cerebral disorder, we have to examine carefully the order of the phenomena, with a view to a correct idea of their origin, nature, and development. In the first place, it is a noticeable general fact that the subjects of all these varied changes in the nervous system are persons already predisposed to the influence of nervine agents. There is either great natural sensibility of that system, or a hereditary tendency to morbid action, or an enfeebled condition of the blood, or (as has not unfrequently happened) medicinal agents have been administered, either by inhalation or potion, with the express intention of inducing this susceptibility; or extreme mental labour has been endured, or a special emotional excitement has been caused.

When the attention of a person thus predisposed is specially roused and directed, the mind receives at the same time a suggestion as to the event likely to result; or, in other words, an *anticipation* is excited. The strenuous act of attention is of itself a powerful modifying agent, and largely exalts the predisposition to irregular nervous action which may already exist, or develops it where it does not. It is therefore a fundamental part of the morbid process that these two acts, *attention* and *anticipation*, be excited. The phenomena will vary almost infinitely, according as they are special or general in their character; that is to say, according as some suggestion is made or particular idea is excited *ab externo*, or according as the direction which the morbid process will take is left to the individual's own mental workings. They are as varied in the former case as the external circumstances from which they originate; in the latter, as the mental constitution, habits of thought, and preconceived ideas upon which they are based.

Amongst the *natural* examples of these monomanias (for they exist as such, and are to be found in all large asylums), the hypochondriacal takes a prominent position. In these, the morbid influence of the attention on the bodily organs has been fully recognised since Sir Henry Holland called special attention to the subject, in the first edition of his "Medical Notes and Reflections." It is generally agreed now by the leading neurologists, that the anticipatory acts of attention which the hypochondriacal patient is constantly directing towards some viscus, is a cause of disease in the viscus involved—not functional only, but structural disease—altering the sensations usually experienced, modifying the mode of action, and finally inducing change in the tissues themselves.

Now, in hypochondriasis, the *anticipation* is of *evil;* the morbid mental state is the *predisposing* source of the suggestion, and the perusal of medical books, or other similar application of the mind, is the *exciting* cause of the *special* ideas upon or from which the mind acts. But there may be anticipation of corporeal *good*, as well as of evil. In this case, the order of the phenomena is still the same—*i. e.*, there is an alteration in the ordinary sensations, a change in the mode of action, and an alteration of structure. To this class of anticipations belong those developed by homœopathy, mesmerism, confidence in treatment, or faith in spiritual influences. These, it is true, result from other sources than those seen in hypochondriasis, but very often indeed the subjects of them are either hypochondriacal or hysterical, or have a predisposition to insanity, or other sensorial disease of the nervous system. To this large group of induced *corporeal* changes belong also the *evil* results consequent upon delusive apprehensions as to the influence of witchcraft, the "evil eye," diabolical agencies, and the like. All morbid acts of attention from these sources, and the morbid anticipations which accompany those acts, may—like those of the hypochondriacal—be followed by both structural and functional changes in the viscera and in the sensations ordinarily connected with them. It is doubtless these results which have so firmly fixed the popular belief in the capabilities for evil of the performers in the black art, and in the power of miraculous gifts of healing professed by members of almost every religious sect in every age, and in every clime. It cannot be denied that persons who profess the power of inflicting injury may become proper objects of punishment if they maliciously and deliberately make the subjects of their malice believe that they are putting those powers into practice, and thereby induce bodily and mental disorder. Indeed, it appears to us that thus to cheat a simple-minded man out of his health and happiness is more criminal than to swindle him out of his property. There are other instances of analogous criminality that might be adduced—*e. g.*, the nefarious methods by which certain advertising quacks act on the mind of their victims, and induce morbid acts of attention and anticipations as to corporeal evil, of the most fearful character.

Perhaps *convulsive movements* of every kind might be properly placed under the last head, when induced by morbidly excited attention. They differ in having a purely cerebral seat, although the phenomena be manifested outwardly. Every form of catalepsy, chorea, and epilepsy are within this group. The monomaniacal societies to which the *dancing* mania of the fourteenth century gave origin may be classed with those of cerebral and intellectual origin, and the disease itself was very similar to some modern affectations not connected (as that was) with religious ideas. Of what we can have no doubt we will, however, more especially speak—namely, those morbid conditions of the sensorial and hemispherical ganglion which a strongly directed attention induces. There are two principal divisions—namely, those in which the mind originates spontaneously the strained attention and the *suggestions* arise in the ordinary way; and those in which the attention is excited artificially, and the suggestions are derived immediately from without. To the former belong all visionaries,*—to the latter, the mesmerized clairvoyantes, the electro-biologized or hypnotized, &c. A third division arises out of a combination of these two sources of morbid action, as in the "evil eye," witchcraft, table-moving, and spirit-rapping. The spectral phenomena which constitute the bases of these forms of mental aberration are by far the most commonly visual, then (in the order of frequency) auditory, muscular,

* Gibbon in his History describes in a few pithy words the process as it was manifested in Peter the Hermit. "Whatever he wished," says Gibbon, "that he believed; whatever he believed, that he saw in dreams and revelations."—Decline and Fall, lviii.

tactile, and olfactory. The points of interest they present are too numerous for consideration here; they also demand, on account of their vast importance, a closer and more critical analysis than is possible at present. It is hardly conceivable to what an extent they have influenced man's conduct through the religious dogmas and political changes that have been based upon them. History is rife with illustrations of their wide-spread influence. In the middle ages, and even later, it was not possible for the wisest men to escape from their influence. The epidemical power of witchcraft may be mentioned as an instance. In numerous instances the accused confessed to circumstances which could only have arisen (if any faith can be placed in common sense) in a morbidly excited state of the system, the notions being precisely those with which the popular mind was imbued from the cradle. We find the following, in Sir W. Scott's "Letters on Demonology and Witchcraft," as to the confessions of a reputed Scotch witch, named Isabel Gouldie:—

"She had been, she said, in the Dounie Hills, and got meal there from the Queen of Fairies, more than she could eat. She added that the queen is bravely clothed in white linen, and in white and brown cloth; that the King of Fairy is a brave man; and there were elf-bulls roaring and skoiling at the entrance of their palace, which frightened her much."

And a great deal more of the same kind.

The *auditory* illusions in these cases have had less attention bestowed upon them than the visual; they are less common, because the sense of hearing has not so extensive relations as the sense of vision, and because the attention is less frequently directed to auditory perceptions, and the *suggestions* which develop them in morbid association are not so numerous. It is in "spirit-rapping" that we have the best modern illustration, although all enthusiasts and visionaries who have received spiritual messages, or held a supposed conversational intercourse with spirits, the Deity, &c., belong to the group. It would, we believe, be altogether contrary to the fact to say that those who profess to hear the "spirit-raps" are deceived by others, or state what they know to be false. They have the strongest possible conviction that the sounds which they hear are genuine, just as in the analogous instance of the insane, and they cannot comprehend by what machinery such distinctly audible sounds can be produced except by agents external to themselves. This conviction is more deeply rooted, when, as it often happens, several persons, being subjected at the same moment to the influence of a common suggestion, and of a morbidly excited attention, hear the same illusory sounds. The following instance shows, however, how these illusions may be induced and removed. A lady being on a visit to a family in which experiments at spirit-rapping were made, became herself a "medium"—that is to say, whenever she touched a door, a table, or a chair, she heard the raps. This was amusing enough for a short time, but on her return home the same circumstance occurred to such an extent that she could touch no article of furniture without hearing these sounds. She wrote to her friends to explain the awkward position she was in, and to beg of them to "exorcise" the spirit—a duty they found to be rather puzzling. However, they wrote back to say that they had taken the necessary steps, and that from the time she received their letter she might confidently rely upon not hearing the spirit any more; and actually from that moment the raps were not heard. We had this history from one of the persons engaged in the transaction, and who has taken great interest in all these phenomena. Of course the reader acquainted with hypnotic or "electro-biological" phenomena will readily recognise the true nature of the "possession" and exorcism. Table-moving is one of the same class, but the *suggestions* direct the muscular system instead of the representative faculty.

Now, amongst the very important moral questions which arise out of the consideration of these phenomena, there is one of transcendent importance—that is, the bearing they have upon the nature and value of *evidence* or *testimony*. There is hardly any more certain conclusion to be drawn from the vast variety of experiments which the psychologist has made to his hands by the numerous classes of persons whose vagaries we have glanced over than this—that sensorial perceptions, ideas, associations of ideas, and general propositions utterly false and erroneous, may be induced by suitable arrangements and manipulations, not only in individuals, but in *large numbers of persons at the same time*, and yet appear to them as being indubitably real and incontrovertibly true. Even when the phenomena are palpably impossible as realities, and the propositions are evidently absurd (as we see in homœopathy, mesmerism, &c.), it is difficult to rebut the evidence thus adduced; but in those cases in which they are probable, although the alleged circumstances did not occur, how can it be *proved* that they are illusions if in themselves not susceptible of re-examination—such as simple spectral illusions and the like? The importance of this question is not diminished when we remember that a designing knave might, by an artful combination of influences, so hypnotize, or otherwise influence the minds of a given number of persons as to make them powerful witnesses in support of a delusion. That such practices constituted part of the system of the sorcerers in the middle and dark ages is, we think, certain; nor are we without well-grounded suspicions that even now, in this the nineteenth century, the nervous system is thus acted upon for the purpose of perpetuating deliberate well-planned frauds. In the "Magie Magnétique" of M. Cahagnet there are several examples of this kind.

What is the remedy for these serious evils? Mr. Faraday has attributed them to defective education in the people, and would improve it in the direction of the Baconian philosophy; but the cultivators of science are themselves the victims of these delusions. As examples, we need go no further than Von Reichenbach and his English translator, the Professor of Chemistry in the University of Edinburgh. The remedy is not easy. The practical psychologist will not, however, lose so favourable an opportunity for the study of the human mind in its aberrations; and the psychiatrician may gather something useful from a careful inquiry into these monomaniacal societies and their literature. The phenomena of which they treat are so closely allied to the hallucinations and notions of the insane, whether we consider their essential nature, their origin, their progressive development, and their results, that for practical purposes they may be considered to be identical. The main difference is in the etiology; in the insane, in the majority of cases, the cause is material rather than mental; in the popular manias, it is rather mental than material.

---

